# Determination of the impacts of supplemental dietary curcumin on post-partum uterine involution using pulsed-wave doppler ultrasonography in Zaraibi goat

**DOI:** 10.1186/s12917-024-04160-2

**Published:** 2024-07-16

**Authors:** Hager Madbouly, K. H. El-Shahat, Elshymaa A. Abdelnaby, Hossam R. El-Sherbiny, Mohamed Fathi

**Affiliations:** Theriogenology Department, Faculty of Veterinary Medicine, Cairo University, Giza, 12211 Egypt

**Keywords:** Curcumin, Doppler, Middle uterine artery, Puerperium, Zaraibi goats

## Abstract

This study aimed to evaluate the impacts of supplemental dietary curcumin on post-partum uterine involution using pulsed-wave Doppler ultrasonography in postpartum goats. Ten pluriparous Zaraibi goats were used and divided into two groups. Group 1 (*n* = 5; control) received only a base diet. Group 2 (*n* = 5; treated) received a base diet supplemented with curcumin (200 mg/kg diet) daily for 28 days, starting from day 1 postpartum (PP) till day 28 PP. Uterine morphometrical changes (uterine horn diameter; UHD and caruncle diameter; CD), uterine hemodynamics (resistance and pulsatility indices (RI and PI), systolic/ diastolic ratio (S/D), peak systolic velocity (PSV), end-diastolic velocity (EDV), blood flow volume (BFV), and blood flow rate (BFR)), and progesterone level were evaluated. Results revealed that the diameter of the uterine horn decreased rapidly from day 1 to day 10 PP (> 50%) but more steadily from day 14 to day 28 PP in both groups. After day 21 PP, there was nearly no reduction in UHD and CD in both groups. The treated group had lower values of the RI and PI (*P* < 0.05) than the control group. Regarding the BFR and BFV in the treated group, there was a significant increase (*P* < 0.05) on day 17 PP, then started to decrease till day 28 PP. While in the control group, there was a significant decrease (*P* < 0.05) in BFR and BFV from day 1 PP till day 28 PP. In conclusion, the incorporation of curcumin in the diet of PP Zaraibi goats improved reproductive performance via improvements in uterine morphometric changes as well as blood perfusion.

## Introduction

The postpartum period is a crucial period that impacts the future fertility of goats [1]. According to Kiracofe [2], uterine involution is necessary for bacterial infection eradication, endometrial histological repair, and proper cyclic activity. Therefore, complete uterine involution is crucial to maintain the following pregnancy [1, 3]. In ruminants, several factors greatly influence uterine involution and ovarian rebound following parturition, such as nutrition, offspring nursing, and the season of delivery [4, 5]. In goats, several studies have indicated different intervals for complete uterine involution. Baru et al. [6] recorded that, on day 19 PP, a complete macroscopic uterine involution was achieved, while Greyling and Van Niekerk [7] reported that complete uterine involution occurred on day 28 PP. Numerous methods were used to examine postpartum uterine involution in goats, including hormonal content analysis, ultrasonography, and morphological examination following slaughter [1, 8]. As the uterus cannot be inspected by rectal or abdominal palpation in small ruminants, ultrasonography is a non-invasive technology that is not only less damaging to dams but also permits intuitive and precise tracking of uterine changes during uterine involution [1, 5]. Alterations in Uterine blood flow have been studied to investigate the progressive changes in the uterus during puerperium [9].

Pregnancy and lactation are the primary causes of oxidative stress in both small ruminants [10] and water buffaloes [11], leading to the formation of reactive oxygen species (ROS) and nitrogen species (RNS), such as nitric oxide radicals (NO), hydrogen peroxide (H2O), superoxide ions, and hydroxyl radicals (OH). These species can cause lipid peroxidation, apoptosis, and infertility [12], and can also affect folliculogenesis, steroidogenesis, and retained placenta, which in turn can impact fertility [13, 14]. Therefore, it is crucial to find a way to protect postpartum goat fertility from the negative effects of ROS. According to earlier research, using antioxidant supplements may reduce oxidative stress and increase fertility [10].

According to Reyes-Gordillo et al. [15], curcumin (CUR) is derived from the rhizomes of the Curcuma longa plant and has the chemical formula 1,7-bis (4-hydroxy-3-methoxyphenyl)-1,6-hepadiene-3,5-dione. This chemical is thought to have a variety of biological actions, including anti-inflammatory, anticancer, antioxidant, antiviral, and antibacterial properties [16]. Inflammation [17], peroxidative damage [18], infection [19], cancer [20], and depression [21] are among the well-established biological, therapeutic, and pharmacological properties of CUR. The antioxidant properties of curcumin include neutralization of superoxide, nitric oxide, and hydrogen peroxide either by eliminating radicals or by boosting the synthesis of catalase (CAT), glutathione peroxidase (GSH-Px), and superoxide dismutase (SOD) [22]. CUR inclusion in the diet of ruminants has been indicated for a variety of purposes, such as enhancing pregnancy outcomes in goats [23, 24] and boosting the reproductive performance of Baladi bucks during the non-breeding season [12]. The purpose of this study was to evaluate the impact of curcumin supplementation on uterine involution through the assessment of uterine morphometrical and hemodynamic changes in postpartum Zaraibi goats.

## Materials and methods

### Ethical approval committee

The study was approved by the institutional animal care committee in the Faculty of Veterinary Medicine, at Cairo University with an approval number: Vet CU 01122022605.

### Experimental animals, feeding and management

The study was conducted at the small ruminant farm of the Faculty of Veterinary Medicine at Cairo University’s Theriogenology Department (30.0276°N, 31.2101°E) from May to July 2022, the temperature during this period was 39 with relative humidity 64%. Ten (*n* = 10) pluriparous post parturient Zaraibi (Egyptian Nubian) goats, aged between 5 and 7 years old, weighing 40 kg on average, were used. All goats had a normal delivery, and the placenta dropped within 8 h after delivery. During the study period, the kids were allowed to suckle normally. The goats were housed in semi-open yards. All the goats were healthy and free of external and internal parasites. All goats were individually fed a daily base diet of 1.5 kg dry matter intake consisting of roughage and concentrate mixture. All goats were fed Egyptian clover (Trifolium alexandrium), green maize (Darawa), and wheat straw as a source of roughage. The concentrate mixture comprised of yellow corn (30%), wheat bran (29%), cottonseed meal (25%), soybean meal (6%), rice bran (4%), molasses (3%), limestone (2%), and common salts (1%). The diet was formulated to meet the nutrient requirements of the NRC [25] for goats.

### Experimental protocol

The goats were divided into two groups. Group 1 (*n* = 5; control) received only a base diet. Group 2 (*n* = 5; treated) received a base diet supplemented with curcumin (Turmeric curcumin, Puritan’s Pride Co., USA; 200 mg/kg ration previously reported by El-Sherbiny et al. [12], Molosse et al. [26] daily for 28 days, starting from day 1 PP till day 28 PP (Day zero = day of parturition). Postpartum uterine involution was monitored in all goats by transrectal ultrasonographic measurement of the diameter of the uterine horn (UHD) and the caruncle diameter (CD) on days 1, 3, 7, 10, 14, 17, 21, 24, and 28 PP.

### Ultrasonography (B-mode and doppler)

**Fig. 1:**
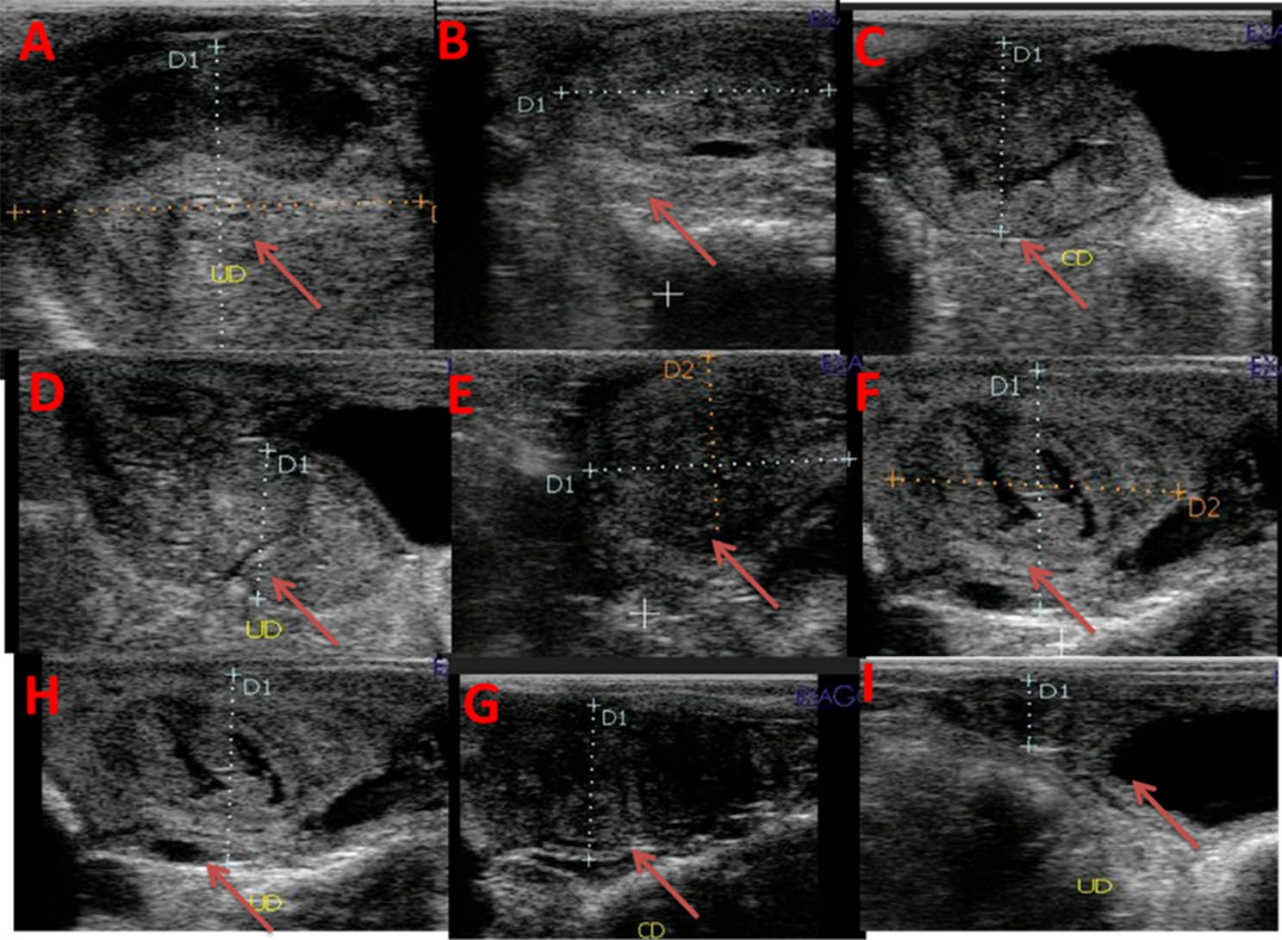
Alterations in the uterine horn diameter at days 1 (**A**), 3 (**B**), 7 (**C**), 10 (**D**), 14 (**E**),17 (**F**), 21 (**G**), 24 (**H**), and 28 (**I**) after parturition in response to curcumin treatment illustrated with red arrows. Uterine horn diameter = UHD in mm

**Fig. 2:**
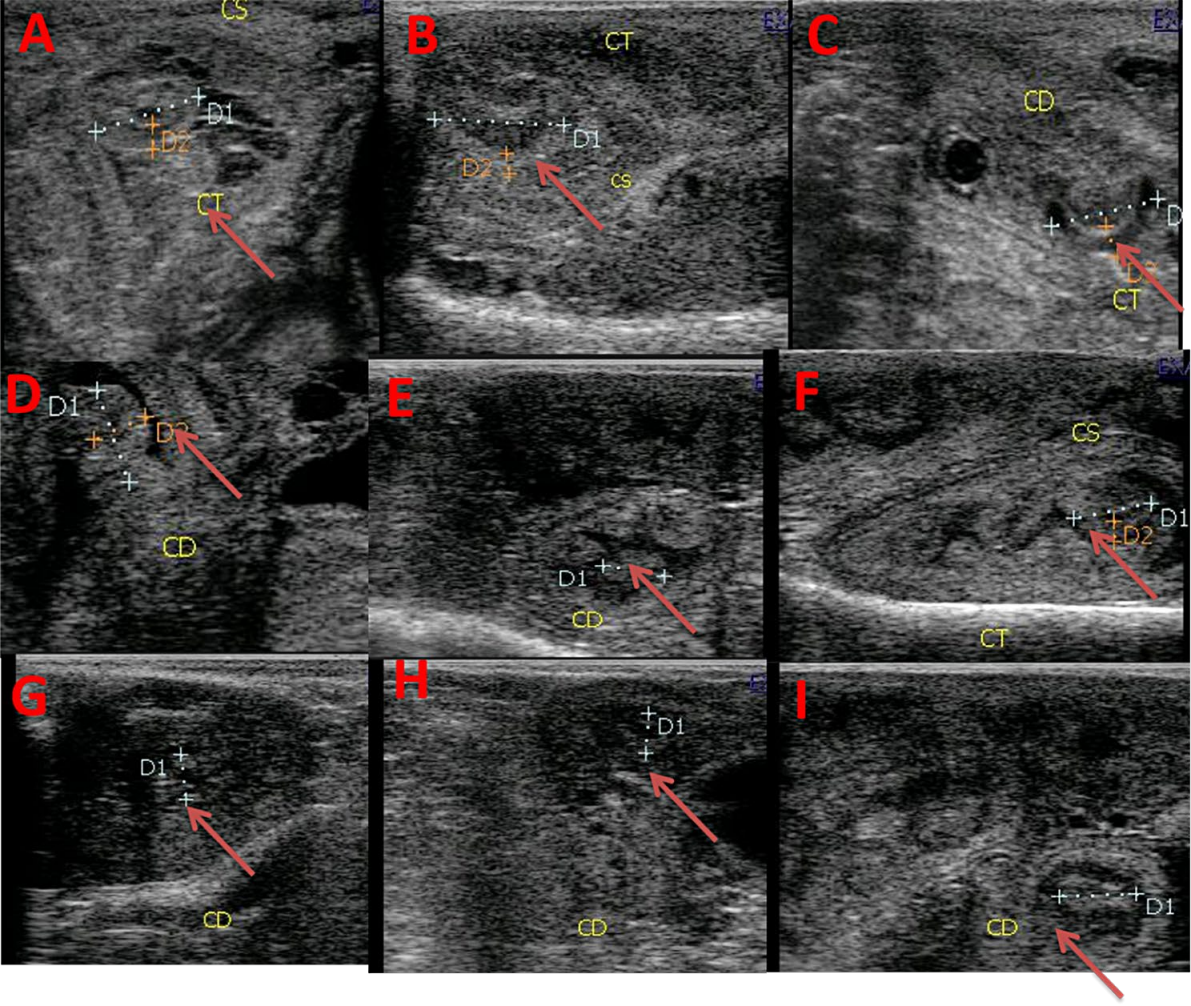
Alterations in the caruncles diameter at days 1 (**A**), 3 (**B**), 7 (**C**), 10 (**D**), 14 (**E**),17 (**F**), 21 (**G**), 24 (**H**), and 28 (**I**) after parturition in response to curcumin treatment illustrated with red arrows. Caruncles diameter = UHD in mm

**Fig. 3:**
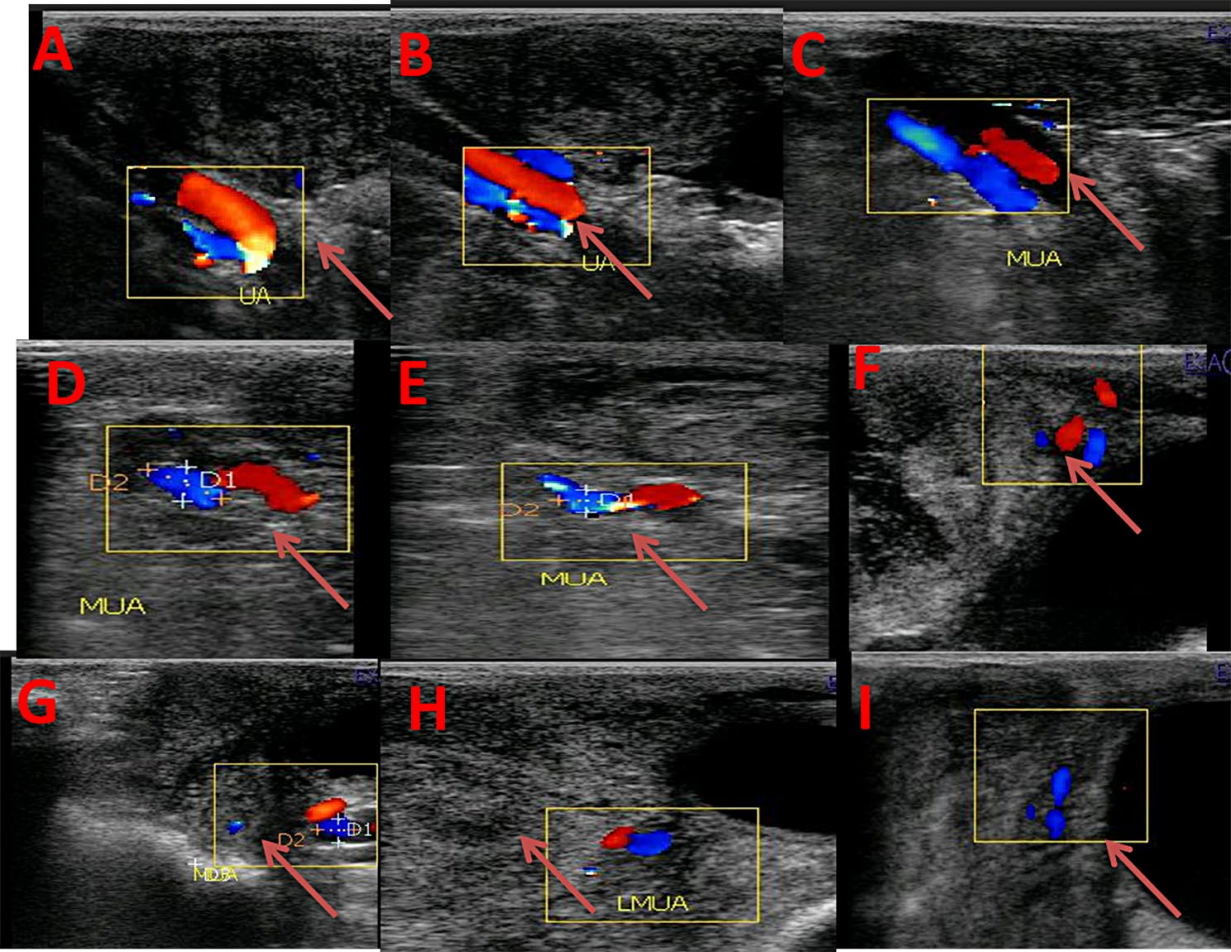
Alterations in the middle uterine artery diameter (MUA diameter/mm) at days 1 (**A**), 3 (**B**), 7 (**C**), 10 (**D**), 14 (**E**),17 (**F**), 21 (**G**), 24 (**H**), and 28 (**I**) after parturition in response to curcumin treatment illustrated with red arrows

Ultrasonographic scanning was performed on days 1, 3, 7, 10, 14, 17, 21, 24, and 28 PP for all goats using a pulsed-wave Doppler ultrasound scanner equipped with a transrectal 5-7.5 MHz linear-array transrectal transducer (EXAGO, Echo Control Medical, made in France). The same operator performed transrectal ultrasonography on the standing goats. After applying ultrasonic gel, the probe was attached to an extension rod and inserted into the emptied rectum. The bladder served as a guide, the transducer was positioned medially and laterally to provide the best view of the uterine horn and caruncle by B mode, and the maximal diameter of the involuting uterine horn (UHD; Fig. 1) was measured [27, 28]. The average diameter of the largest three to five caruncles was utilized to estimate the caruncle diameters (CD; Fig. 2) in B mode image. Uterine involution was complete when there was no additional reduction in uterine diameter for three consecutive examinations; the absence of lochia in the uterus and caruncles were difficult to visualize [1]. The middle uterine artery (MUA; Fig. 3) in a PP goat was assessed transrectally in the standing position. After detecting the urethral artery that supplies the urinary bladder, then uterine artery was merged from the internal iliac artery, and the flow was recorded as demonstrated by Elmetwally et al. [29]. The color flow mode of Doppler ultrasound was switched on to locate the middle uterine artery. When the uterine artery was located cranio-lateral to the bladder and near the external iliac artery, the spectral Doppler mode was activated with a gate width of 1 mm, a maximum velocity of 35 cm/s, and an angle of insonation of 40° [30, 31]. The hemodynamic parameters of the middle uterine artery, including resistance and pulsatility indices (RI and PI), systolic/ diastolic ratio (S/D), peak systolic velocity (PSV), end-diastolic velocity (EDV), blood flow volume (BFV), and blood flow rate (BFR), were measured by the device.

### Blood sampling and progesterone assay

Blood samples (5 mL) were collected on the same days of the ultrasound examination from the jugular vein puncture into plain vacutainer tubes and centrifuged at 3000 rpm for 15 min. Then, serum samples were stored at -20 ° until assessment. Progesterone (EL 1-1259 –lotPGS5753-96 well) was measured using competitive enzyme-linked immunosorbent assay kits (Monocent, Inc., USA). The sensitivity of the test was 0.112 ng/mL.

### Statistical analysis

The data was presented as mean ± standard error of the mean [23]. The data were analyzed using SPSS software version 20 (Microsoft Corp. 1984–2000 Inc.), with one way and two-way ANOVA options including a general linear model to demonstrate changes in each variable across all time points studied in addition to the interaction between time and treatment. Duncan’s multiple range test was applied to identify a significant difference with a probability of less than 0.05.

## Results

### Post-partum uterine findings during dietary curcumin supplementation

#### B- mode evaluation of postpartum uterine involution in goats

**Fig. 4:**
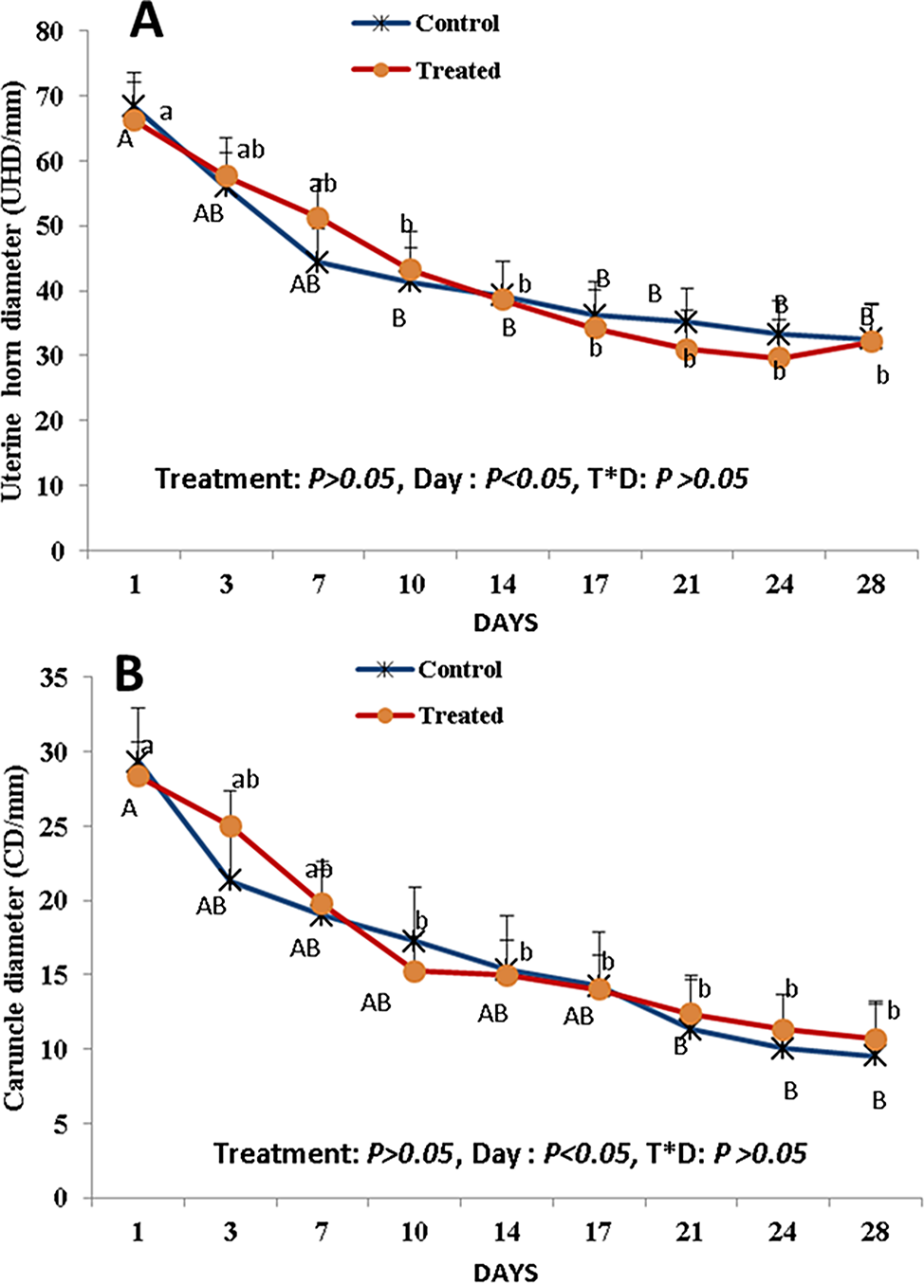
Alterations in the uterine horn diameter (UHD; mm; A) and the caruncle diameter (CD; mm; B) from day 1 till day 28 after parturition. Capital letter (**A** and **B**) superscripts values are significantly different at *P* < 0.05 in control goats along days of examination, while small letters (**a** and **b**) superscripts values are significantly different at *P* < 0.05 in curcumin-treated goats along days of examination. There was no effect of treatment on UHD or CD during the days of the examination. There was no interaction between treatment and time for those parameters

The diameter of the uterine horn decreased rapidly from day 1 to day 10 PP (> 50%) but more steadily from day 14 to day 28 PP in both the curcumin-treated and control groups (Fig. 4A). Caruncle diameter (CD) significantly decreased (*P* < 0.05) in the control group between day 1 and day 21 PP, but in the treatment group, CD significantly differed (*P* < 0.05) between day 1 and day 10 PP (Fig. 4B). After day 21 PP, there was nearly no reduction in UHD and CD in both groups, suggesting that the end of uterine involution occurred at approximately 21 days PP. There was no effect of treatment on UHD or CD during the days of examination. There was no interaction between treatment and time for those parameters.

#### Pulsed-wave doppler results for uterine artery in postpartum goats

**Fig. 5:**
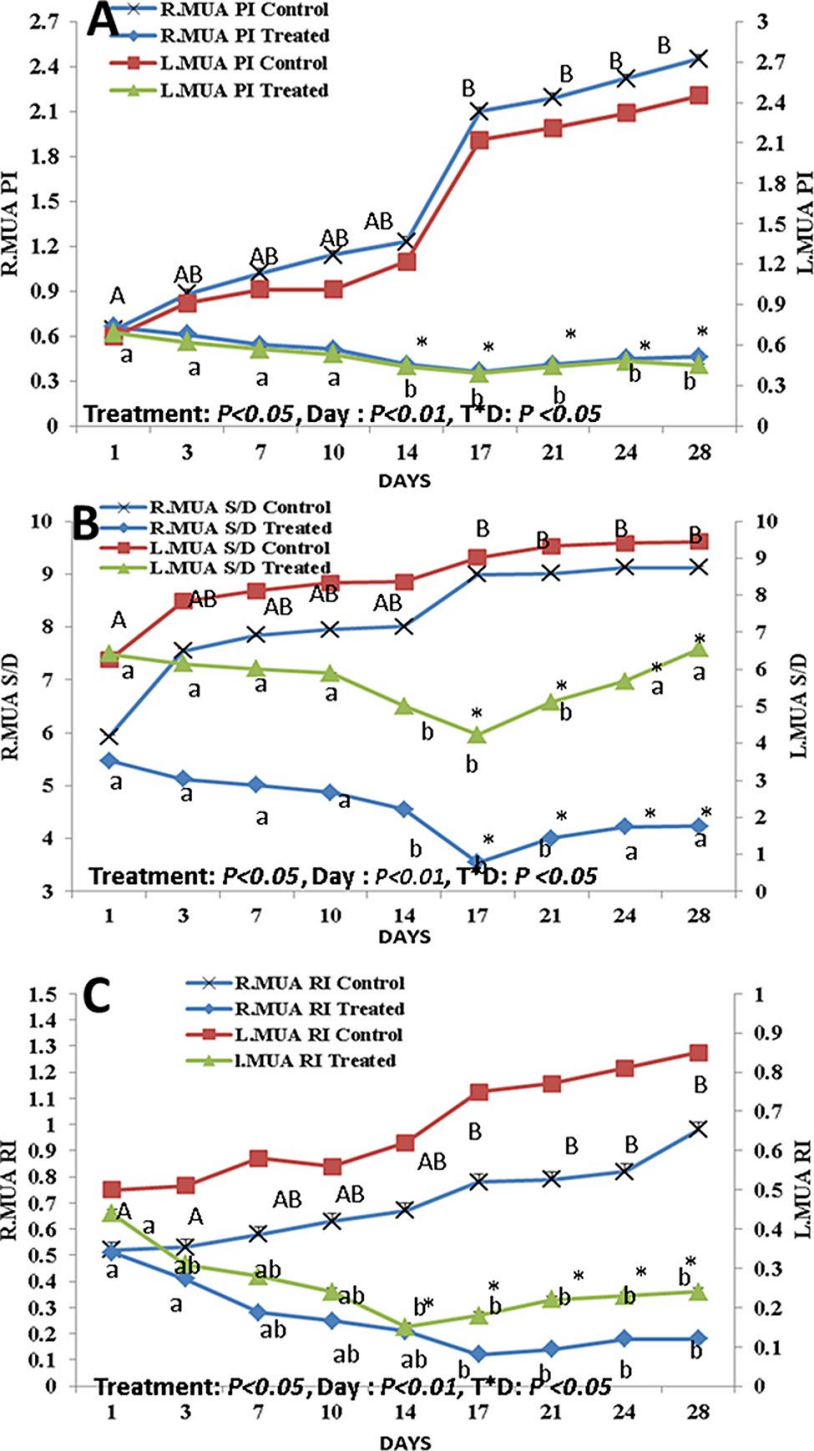
Alterations in the middle uterine artery pulsatility index (MUA PI; **A**), systolic/diastolic (MUA S/D; **B**), and resistance index (MUA RI; **C**) on both right and left sides from day 1 till day 28 after parturition. Capital letter (A and B) superscripts values are significantly different at *P* < 0.05 in control goats along the days of examination, while small letters (a and b) superscripts values are significantly different at *P* < 0.05 in curcumin treated goats along days of examination. * Means the two groups are significantly different at *P* < 0.05 at the same time point. There was a treatment effect and day effect between both groups at *P* < 0.05 and *P* < 0.01 respectively, in addition an interaction was present between day and treatment at *P* < 0.05

**Fig. 6:**
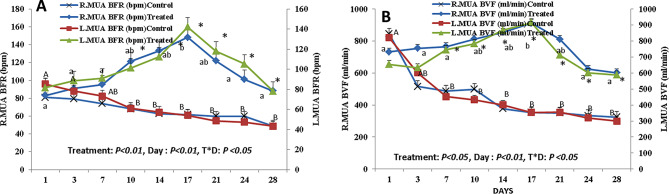
Alterations in the middle uterine artery blood flow rate (MUA BFR [bpm]; **A**) and blood flow volume (MUA BFV [ml/min]; **B**) on both right and left from day 1 till day 28 after parturition. Capital letter (**A** and **B**) superscripts values are significantly different at *P*<0.05 in control goats along days of examination, while small letters (**a** and **b**) superscripts values are significantly different at *P*<0.05 in curcumin treated female goats along days of examination. * Means the two groups are significantly different at *P*<0.05 at the same time point. There was a treatment effect and day effect between both groups at (*P*<0.01 for BFR, *P*<0.01 for BFV) and *P*<0.01 for both parameters, respectively. In addition, an interaction was present between the day and treatment at *P*<0.05

The effect of curcumin supplementation on uterine blood flow is shown in Figs. 5 and 6. There was a treatment effect and a day effect on the Doppler indices (RI, PI, and S/D ratio) of the middle uterine artery between both groups at *P* < 0.05 and *P* < 0.01, respectively. In addition, an interaction was present between the day and treatment at *P* < 0.05. The treated group had lower values of the RI and PI (*P* < 0.05) than those of the control group (Fig. 5A and C). In the control group, there was a significant increase (*P* < 0.05) in the S/D ratio from day 17 PP until day 28 PP. While in the treated group, there was a significant decrease (*P* < 0.05) in the S/D ratio on days 14, 17, and 21 PP, with the lowest value on day 17 PP (Fig. 5B). Regarding the BFR and BFV, there was a treatment effect and a day effect between both groups at (*P* < 0.01 for blood flow rate and blood flow volume). In addition, an interaction was present between the day and treatment at *P* < 0.05 (Fig. 6). In the treated group, there was a significant increase (*P* < 0.05) in BFR and BFV on day 17 PP, then started to decrease till day 28 PP (Fig. 6A and B). While in the control group, there was a significant decrease (*P* < 0.05) in BFR and BFV from day 1 PP till day 28 PP (Fig. 6A and B).

### Progesterone profile in postpartum goats

**Fig. 7:**
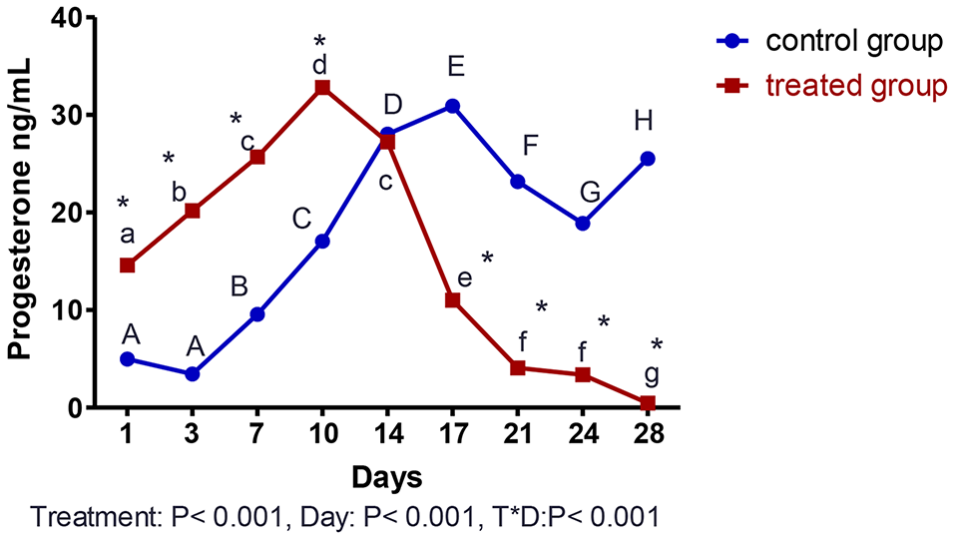
Alterations in serum levels of progesterone (ng/mL) from day 1 till day 28 after parturition. Capital Letters (A,B,C,D,E,F,G and H) superscripts values are significantly different at *P* < 0.001 in control goats along days of examination, while small letters (a, b, c, d, e, f, and g) superscripts values are significantly different at *P* < 0.001 in curcumin treated goats (Treated) along days of examination. * Means the two groups are significantly different at *P* < 0.001 at the same time point. There was a treatment effect and day effect between oth groups at *P* < 0.001, and in addition, an interaction was present between day and treatment at *P* < 0.001

The effect of curcumin supplementation on serum P4 levels from day 1 to day 28 PP is shown in Fig. 7. There was a treatment effect and a day effect between both groups at *P* < 0.001. Furthermore, an interaction was present between the day and treatment at *P* < 0.001. In the treated group, there was a significant decrease (*P* < 0.001) in the P4 level on day 1 PP, then started to increase till reaching a peak level on day 10 PP, then a sudden fall in the P4 level occurred from day 14 PP till reaching the lowest level on day 28 PP (Fig. 7). In the control group, there was a significant decrease (*P* < 0.001) in the P4 level on days 1 and 3, then started to increase until it reached its highest level on day 17 PP. After that, the P4 level decreased on days 21 and 24 PP, then elevated again on day 28 PP. There was a significant decrease in P4 level (*P* < 0.001) in the treated group compared to the control group from day 17 to day 28 PP (Fig. 7).

## Discussion

There are few studies on Doppler-guided PP uterine involution in small ruminants, and most of them are based on postmortem examination [7, 8]. B-mode ultrasonography was used in examination uterine changes [1, 32–34], but the introduction of Doppler ultrasound is very critical in veterinary reproduction [35]. This study was intended to investigate, by pulsed-wave Doppler sonography, the involution of the goat uterus in the PP period following curcumin supplementation in the diet. Although curcumin has been widely used in several animal models to alleviate a variety of stress conditions and improve their performance [23, 24, 36], its use for improving the reproductive performance of the Zaraibi goats in the PP period has not yet been studied. The results of the present study support the hypothesis that dietary supplementation with curcumin influences the Zaraibi goats’ reproductive performance, as evidenced by improvements in uterine blood perfusion, which will be reflected in the resumption of the reproductive cycle and goat reproduction.

In the present study, there was a rapid decrease in UHD from day 1 to day 10 PP (> 50%). This result agreed with the findings of Badawi et al. [5], who found a rapid decrease in uterine diameter (˃50%) between day 3 to day 14 PP in Nubian goats. According to studies on sheep, uterine size decreased by more than 50% in Farafra sheep during the first two weeks of delivery [28]. This finding differs from that of German sheep [33], who claimed that over 80% of uterine involution occurred within the first 11 days of PP. Degefa et al. [8] reported that uterine regression passed through three stages and was completed by Days 7 PP, 13 PP, and 19 PP, respectively. Furthermore, Zongo et al. [3] pointed out that complete uterine involution in goats occurred between days 18 and 22 PP. Ababneh and Degefa [32] also found that most of the uterine involution in Balady goats commenced within one week of PP. This study revealed that the end of uterine involution occurred approximately 21 days after PP, which was characterized by a small UHD (no notable changes in UHD for three consecutive examinations were recorded). This finding is consistent with previous research by Ababneh and Degefa [32], Takayama et al. [37], Badawi et al. [5] but contrasts with the observations of Rubianes and Ungerfeld [38], Zdunczyk et al. [1], and Hayder and Ali [28], who reported the end of uterine involution at 30 days PP. A shorter (23 days) pp period was previously reported in Nubian goats by Makawi and Badawi [39]. In addition, longer PP periods were reported in Boer [40], Anglo-Nubian, Saanen [41], Somali [42], and Nilotic goats [43]. These variations in the duration of the PP period may be due to differences among breeds, measurement methods, treatments in the PP period, and the influence of seasons [40]. In this study, the RI and PI values of the MUA in the curcumin treated group were considerably lower than those of the control group, indicating a decrease in blood vessel impedance and higher uterine blood perfusion and functions. Also, there was a significant increase in BFR and BFV on day 17 PP in the curcumin treated group in addition to the inverse relation between both Doppler indices and blood flow velocity [44]. While in the control group, there was a reduction in both BFR and BFV of the MUA during the postpartum period. Similarly, Elmetwally and Bollwein [9] observed a significant decrease in uterine blood flow over the first nine days of PP, with a 70% reduction in blood flow volume on day 6. On the other hand, the improvement in uterine hemodynamics observed in curcumin-treated goats may be attributable to the reduction of oxidative stress-mediated vascular endothelial dysfunction through its significant antioxidant properties [45]. This was achieved through ROS (especially superoxide anion) capturing and increasing bioavailable nitric oxide (a potent vasodilator). Moreover, the endothelial nitric oxide synthase enzyme (eNOS), which is accountable for acting on arginine for NO biosynthesis, could be deactivated (uncoupled) through ROS-mediated arginase enzyme activation, which resulted in decreased blood flow and NO bioavailability [46]; therefore, the antioxidant capabilities of curcumin are thought to be the key reason for the improvement in uterine blood perfusion. This result agreed with the findings of El-Sherbiny et al. [12], who reported that curcumin could improve testicular hemodynamics via its antioxidant effect during the non-breeding season in Baladi bucks. The negative energy balance around the periparturient period, particularly the first week after giving birth, may lead to an increase in the mobilization of fat. This process is related to the generation of lipid peroxides and reactive oxygen species (ROS) and increased levels of SOD in the early puerperium [47]. When ROS production outpaces the capacity of biological systems to remove these reactive molecules, it leads to oxidative stress [48]. Therefore, antioxidants that provide nutrition may be necessary to effectively reduce oxidative stress in the PP period [49]. In this work, the antioxidant properties of curcumin played a role in improving uterine blood perfusion, which is expected to be reflected in the resumption of ovarian activity and goat reproduction. In the present study, the progesterone level gradually increased until reaching a peak level on day 10 PP in the treated group, which might be a result of the resumption of postpartum ovarian cyclicity. This result agreed with Hussain et al. [50], who found that progesterone concentration stayed at basal levels in the PP anestrous period and displayed an increase with the resumption of PP cyclicity in goats.

## Conclusion

It could be concluded that the incorporation of curcumin in the diet of postpartum Zaraibi goats improved reproductive performance via improvements in uterine blood perfusion and uterine morphometric changes. Therefore, curcumin supplementation in the diet of PP goats can be a promising approach for improving goat fertility.

## Data Availability

No datasets were generated or analysed during the current study.
